# Functional Responses and Resilience of Boreal Forest Ecosystem after Reduction of Deer Density

**DOI:** 10.1371/journal.pone.0090437

**Published:** 2014-02-28

**Authors:** Marianne Bachand, Stéphanie Pellerin, Marco Moretti, Isabelle Aubin, Jean-Pierre Tremblay, Steeve D. Côté, Monique Poulin

**Affiliations:** 1 Chaire de recherche industrielle CRSNG-Produits Forestiers Anticosti, Département de biologie, Université Laval, Québec, Québec, Canada; 2 Centre d'études nordiques, Université Laval, Québec, Québec, Canada; 3 Québec Centre for Biodiversity Science, McGill University, Montréal, Québec, Canada; 4 Institut de recherche en biologie végétale, Jardin Botanique de Montréal, Montréal, Québec, Canada; 5 Swiss Federal Research Institute WSL, Bellinzona, Switzerland; 6 Great Lake Forestry Centre, Canadian Forest Service, Sault-Sainte-Marie, Ontario, Canada; Brigham Young University, United States of America

## Abstract

The functional trait-based approach is increasingly used to predict responses of ecological communities to disturbances, but most studies target a single taxonomic group. Here, we assessed the resilience of a forest ecosystem to an overabundant herbivore population by assessing changes in 19 functional traits for plant, 13 traits for ground beetle and 16 traits for songbird communities after six years of controlled browsing on Anticosti Island (Quebec, Canada). Our results indicated that plants were more responsive to 6 years of reduced browsing pressure than ground beetles and songbirds. However, co-inertia analysis revealed that ground beetle communities responded in a similar way than plant communities with stronger relationships between plant and ground beetle traits at reduced deer density, a pattern not detected between plant and songbird. High deer density favored plants species that reproduce vegetatively and with abiotic pollination and seed dispersal, traits implying little interaction with animal. On the other hand, traits found at reduced deer density mostly involved trophic interaction. For example, plants in this treatment had fleshy fruits and large seeds dispersed by birds or other animals whereas ground beetle species were carnivorous. Overall, our results suggest that plant communities recovered some functional components to overabundant herbivore populations, since most traits associated with undisturbed forests were reestablished after six years of deer reduction. The re-establishment of functional plant communities with traits involving trophic interaction induces changes in the ground-beetle trait community, but forest structure remains likely insufficiently heterogeneous to shift the songbird trait community within six years.

## Introduction

Predicting the response of communities to environmental changes is a fundamental issue in ecosystem ecology. Recently, there has been a growing interest in the use of functional traits to identify the mechanisms that underlie community changes and determine ecosystem functioning [1], [2]. Functional traits refer to any morphological, anatomical, biochemical, physiological or phenological features associated with a species' ability to obtain resources, disperse, reproduce and persist in the environment [3]. An approach based on functional traits offers several advantages. For instance, it facilitates comparisons between communities that do not share the same species composition [4], and provides insight into the processes governing community structure and ecosystem services [5].

Most studies using a functional trait-based approach have focused on a single taxonomic group (for example, [6], [7]). Yet, there are growing expectations that extending the trait concept to multiple taxonomic groups will improve our ability to understand the complex dynamic of ecosystems and identify mechanisms that drive biotic control over ecosystem functions [8], [9], [10], [11]. The distinction between response and effect traits has been proposed as a core element of a multi-taxa assessment [2], [12]. Researchers have typically been interested in response traits (i.e., traits explaining a species' response to a given environmental gradient). However, a response-and-effect trait framework makes it possible to characterize both (1) how a community responds to an environmental filter (response traits); and (2) how this community shift might in turn influence a given ecosystem process via effect traits. Here, we examine the effect of reducing large herbivore browsing pressure on community-level functional response and effect traits using a multiple taxonomic groups approach. We focused on plants, ground beetles and songbirds, each taxon with different mobility capacity and type of resources uses, characteristics expected to strongly influence response to environmental changes.

The overabundance of large ungulates has tremendous effects on forest resources in many regions worldwide [13]. For example, selective browsing by ungulate herbivores induces the disappearance of preferred species, leads to the dominance of avoided or browse-resilient species [14], [15] and may prevent tree and shrub regeneration [16], [17]. Such changes may later indirectly affect key ecosystem processes such as nutrient cycling, soil mineralization, and litter quality [18], [19]. Indirect impacts on other biological organisms, especially birds and insects, have also been observed, and are mostly related to changes in habitat structure [20], [21], [22], [23]. Some studies have also shown that heavy browsing favors specific plant traits such as abiotic pollination and long distance seed dispersal [24], [25], which involve no biotic interaction and may thereby induce the decline of animals dependent on flowers and fruits.

Most studies on the impacts of ungulate browsing pressure have focused on plants, comparing *in situ* densities with areas where herbivores have been excluded or reduced (for example, [26], [27]). Empirical studies using traits of more than one taxonomic group with different levels of herbivore density reduction would be highly beneficial for understanding ecosystem capacity to recover once browsing pressure has been reduced. In this study, we used a multi-factorial controlled browsing experiment conducted over six years to investigate the direct impact of reducing white-tailed deer (*Odocoileus virginianus*) density on plant communities and the indirect effect on animal communities through vegetation changes. Our objectives were to define specific functional syndromes (i.e., consistent association of traits) of different taxonomic groups (plants, ground beetles and songbirds) and highlight the cascade effect across taxa generated by reducing deer density in a boreal forest. We hypothesized that the effects of reduced deer density would be stronger for plants, since they are directly affected by browsing. We further expected to find that changes in the effect traits among the plant community would in turn affect ground beetles and songbirds. In this regard, we anticipated weaker relations across taxa (plants-ground beetles and plants-songbirds) at high deer density than with reduced ungulate presence.

## Materials and Methods

### Ethics Statement

Animal handling protocols were approved by the Université Laval Animal Care Committee of the Canadian Council on Animal Care (UL 2008017-1). Our experiment complies with the laws of Canada and to accepted international ethical standards.

### Site Description

The study was carried out on Anticosti Island (7943 km^2^) in the Gulf of St. Lawrence (Québec, Canada; 49° 28′ N; 63° 00′ W). Climate is maritime and characterized by cool summers and long but relatively mild winters [28]. The island is located about 70 km north of the natural northeastern limit of the white-tailed deer distribution range. About 220 white-tailed deer were introduced in 1896–97, the first large ungulate on this predator-free island. The population proliferated, becoming overabundant (>20 deer · km^−2^) in less than 30 years (Potvin et al. 2003). Anticosti's forests belong to the boreal zone, and were naturally dominated by balsam fir (*Abies balsamea*), white spruce (*Picea glauca*) and black spruce (*Picea mariana*), with deciduous tree species, mainly paper birch (*Betula papyrifera*), trembling aspen (*Populus tremuloides*) and balsam poplar (*Populus balsamifera*) occurring sporadically. Despite the short history of deer herbivory on the island, the impacts of deer browsing on the composition and dynamics of its forest ecosystems are extensive. For instance, the surface area covered by balsam fir stands, a key habitat for deer winter survival, has been reduced by half over the last hundred years and replaced by white spruce stands [29].

### Experimental Design

Our study utilized the infrastructure of a long-term experiment that was initiated in 2001 and designed to investigate the impact of reducing deer density on the reproduction and growth of plants in two types of vegetation cover: uncut forests and cut-over areas. This experimental set-up is a full factorial strip-plot design with main plots replicated in three complete blocks (located between 4 and 71 km apart). Each block is composed of four main plots (adjacent or in close proximity within each block). They consist of three large enclosures with distinct deer densities (0, 7.5, 15 deer · km^−2^) and a control situation outside the fence (*in situ* densities: 27, 56 and 56 deer · km^−2^). To control deer density, all deer were removed from all enclosures each year. No deer were reintroduced in a 10-ha enclosure (0 deer · km^−2^), whereas three deer were stocked in each of the two other enclosures, one measuring 40 ha (7.5 deer · km^−2^) and the other 20 ha (15 deer · km^−2^). Deer (yearlings or adults) were captured in early spring, released within enclosures and culled in late autumn. Deer enclosures were closely monitored to detect and subsequently repair any broken fences, and thereby impede intruders as well as deer escape, injury or fatality. Deer stocking began in 2002 and was repeated annually until 2009. The *in situ* deer densities were monitored on unfenced sites using distance sampling of summer pellet groups on permanent transects cleared of feces each spring [27]. In each main plot, all trees >9 cm at breast height had been removed over about 70% of the area, leaving about 30% of the mature balsam fir forest (mean size of uncut forest patches was 5.9±8.2 ha). Cut-over was included in the design because it has been used on Anticosti as a catalyst to stimulate balsam fir regeneration since 1995 [30]. Both types of vegetation cover were characterized by >70% balsam fir canopy cover before the beginning of the experiment.

### Sampling

Three taxonomic groups with different mobility capacity and resource use patterns were selected as model groups for our study: (1) plants, which have low mobility (for escaping herbivory) and are directly affected by deer; (2) ground beetles, with low to moderate mobility and indirectly affected by deer, mostly via changes in vegetation composition and ground surface conditions at a local scale (i.e., a few square meters); and (3) songbirds, with high mobility and indirectly affected by deer through changes in forest structure at a moderate-large spatial scale (i.e., hundreds of square meters or more). Sampling was conducted during summer 2007, thus six years after the establishment of the experimental set-up.

#### Plants

Plants were sampled within 20 permanent quadrats (10×10 m) that had been randomly positioned in 2001 in each of the 12 main plots on both vegetation cover types (*N* = 480 quadrats). Each quadrat was subdivided into 100 subquadrats of 1×1 m, two of which were randomly selected for surveys. In each subquadrat, the horizontal cover of each vascular plant species (used as abundance data) was estimated according to 12 classes (<1, 1–5, 10 classes up to 95, 95–100%). Cover of trees and shrubs taller than 2.5 m were not surveyed because they were inaccessible to deer and because subquadrat size was inappropriate for these vegetation layers.

#### Ground beetles

Ground beetles were surveyed by Brousseau et al. (2013) [23], using luminous traps [31] as pitfall traps to attract a large diversity and abundance of beetles [32]. In each of the 12 main plots, two pitfall traps were installed in each vegetation cover type and an internal recipient was filled with 40% ethyl alcohol as a preservative (*N* = 48 traps). Traps were placed at least 50 m apart, 100 m away from fences and, wherever possible (i.e., when the forest patch was sufficiently large), at least 50 m from forest edges. Traps were operated during five periods of 9 to 11 consecutive days between mid-June and mid-August (the main activity period for ground dwelling arthropods in the region), for a total of 50 trap-days in each experimental unit. Abundance of the insect taxa was defined as the number of individuals found in the trap within the sampling period.

#### Songbirds

The relative abundance of songbirds was surveyed by Cardinal et al. (2012) [22] in 2007, using point counts during the nesting period [33]. In each of the 12 main plots, two point count stations were centered on randomly selected uncut forest patches, and three stations were located randomly in cut-over areas (*N* = 60 stations). The stations were separated by at least 100 m. The difference in number of points between uncut forests and cut-over areas results from the smaller proportion of uncut forests (30%) compared to the cut-over areas (70%) in each main plot. A 50-m buffer zone was maintained from fence or forest edges to avoid edge effects. Individual songbirds were counted for each species heard and seen over a period of 20 minutes at each point-count. Point-counts were visited six times from June 5 to 30, between 4:30 and 10:00 am, always under favorable weather conditions, i.e., without rain or strong winds. We defined the abundance of songbird species at each point-count as the highest count of individuals of a given species among all visits at that station during the sampling season.

### Species Traits

Each species was described in terms of its traits, including both traits related to morphology, reproduction and dispersal, as well as performance traits related to resource use [3]. For plants, we also used the status (exotic or indigenous), since exotic species are often associated with disturbed environments. We selected 19 traits for plants (Table 1), 13 for ground beetles (Table 2) and 16 for songbirds (Table 3). Plant traits were obtained from the TOPIC database for plants (http://topic.rncan.gc.ca). For ground beetles and songbirds, we used traits from the MultiTraits database (Venier et al. Unpublished data). To meet statistical analysis assumptions, all traits were coded numerically (Tables 1–3).

**Table 1:** Description and codes of the plant functional traits used in this study.

Trait	Code	Type	Trait unit
**Morphology**			
Foliage persistence	PFO	Binary	0. deciduous, 1. evergreen
Foliage structure	SFO	Ordinal	0. no leaf, 1. rosette, 2. graminoid, 3. erect leaves, 4. decumbent, 5. erect leafy stem, 6. one stem, 7. multi-stem
Raunkiaer life form	RAU	Ordinal	1. therophyte, 2. geophyte, 3. hemicryptophyte, 4. chamaephyte, 5. micro/nano phanerophyte, 6. mega/meso phanerophyte
Rhizome	RHI	Binary	0. absence, 1. presence
Storage organ	STO	Binary	0. absence, 1. presence
Physical defense	DEF	Binary	0. absence, 1. presence
Life cycle	LCY	Ordinal	1. annual, 2. biannual, 3. perennial
**Reproduction and dispersal**			
Principal means of reproduction	VEG	Ordinal	1. seeds only, 2. vegetative propagation possible but mostly by seeds, 3. mostly by vegetative propagation
Inflorescence type	TFL	Ordinal	1. no flower, 2. solitary, 3. spike or cluster, 4. composed
Inflorescence color	CFL	Ordinal	1. no flower, 2. green, brown and black, 3. white, 4. other colors
Flowering phenology:			
Spring	SPR	Binary	0. absence, 1. presence
Summer	SUM	Binary	0. absence, 1. presence
Fall	FAL	Binary	0. absence, 1. presence
Cleistogamy	CLE	Binary	0. absence, 1. presence
Pollinator vector:			
Abiotic	POA	Binary	0. absence, 1. presence
Biotic	POB	Binary	0. absence, 1. presence
Self-pollination	PON	Binary	0. absence, 1. presence
Seed size	SES	Quantitative	Millimeter
Seed production	SEP	Quantitative	Seed (number of)
Seed dispersal vector:			
Wind	WIN	Binary	0. absence, 1. presence
Endozoochore	END	Binary	0. absence, 1. presence
Epizoochore	EPI	Binary	0. absence, 1. presence
Ant	MYR	Binary	0. absence, 1. presence
Bird	BIR	Binary	0. absence, 1. presence
Expulsion	BAL	Binary	0. absence, 1. presence
Gravity	GRA	Binary	0. absence, 1. presence
Dispersal type	DIT	Ordinal	1. spore, 2. dried fruit, 3. fleshy fruit
**Resource use**			
Light requirement	HEL	Ordinal	1. shade tolerant, 2. mid tolerant, 3. intolerant
**Status**			
Status	STA	Binary	1. indigenous, 2. exotic

**Table 2:** Description and codes of ground beetle functional traits used in this study.

Trait	Code	Type	Trait units
**Morphology**			
Body size	BOD	Quantitative	Millimeter
Defense mechanism:			
Chemical	DCH	Binary	0. absence, 1. presence
Physical	DPH	Binary	0. absence, 1. presence
Sound	DSO	Binary	0. absence, 1. presence
Number of months active by year	NMA	Quantitative	Month (number of)
**Reproduction and dispersal**			
Wing condition	WIG	Ordinal	1. brachypterous, 2. dimorphic, 3. macropterous
Flying habit	FLY	Ordinal	1. incapable, 2. occasional, 3. frequent
Hibernation form	HIB	Ordinal	1. larva, 2. adult and larva, 3. adult
**Resource use**			
Light requirement	HEL	Ordinal	1. shade tolerant, 2. mid tolerant, 3. intolerant
Moisture Level	XER	Ordinal	1. moist, 2. mesic, 3. dry
Feeding guild:			
Carnivorous	CAR	Binary	0. no, 1. yes
Granivorous	GRN	Binary	0. no, 1. yes
Insectivorous	INS	Binary	0. no, 1. yes
Omnivorous	OMN	Binary	0. no, 1. yes
Vertical stratification	VST	Ordinal	1. terricolous, 2. arboreal and terricolous
Habitat structure	CLO	Ordinal	1. open, 2. generalist, 3. closed
Type of vegetation	TVE	Ordinal	1. devoid of vegetation, 2. sparse herbaceous, 3. moderate herbaceous, 4. dense herbaceous
Substrate:			
Clay	CLA	Binary	0. no, 1. yes
Gravel	GRV	Binary	0. no, 1. yes
Humus	HUM	Binary	0. no, 1. yes
Leaf litter	LEA	Binary	0. no, 1. yes
Moss	MOS	Binary	0. no, 1. yes
Sand	SAN	Binary	0. no, 1. yes

**Table 3:** Description and codes of songbird functional traits used in this study.

Trait	Code	Type	Trait value in statistical analyses
**Morphology**			
Wing length	WIL	Quantitative	Millimeter
Tarsus length	TAR	Quantitative	Millimeter
Weight	WEI	Quantitative	Gramm
**Reproduction and dispersal**			
Nest substrate:			
Cliff	NCL	Binary	0. no, 1. yes
Ground	NGR	Binary	0. no, 1. yes
Shrub	NSH	Binary	0. no, 1. yes
Deciduous	NDE	Binary	0. no, 1. yes
Coniferous	NCO	Binary	0. no, 1. yes
Mixed tree	NTR	Binary	0. no, 1. yes
Snag	NSN	Binary	0. no, 1. yes
Nest height	NHG	Quantitative	Meter
Nest form:			
Cup	CUP	Binary	0. no, 1. yes
Cavity	CAV	Binary	0. no, 1. yes
Burrow	BUR	Binary	0. no, 1. yes
Pendant	PEN	Binary	0. no, 1. yes
Spherical	SPH	Binary	0. no, 1. yes
Clutch size	CLU	Quantitative	Egg (number of)
Number of broods	BRO	Quantitative	Brood (number of)
Nest parasited by cowbird	COW	Quantitative	Nest (number of)
**Resource use**			
Primary habitat:			
Deciduous	DEC	Binary	0. no, 1. Yes
Coniferous	CON	Binary	0. no, 1. Yes
Mixed forest	MIX	Binary	0. no, 1. Yes
Agricultural	AGR	Binary	0. no, 1. Yes
Wetland	WEA	Binary	0. no, 1. Yes
Bog	BOG	Binary	0. no, 1. Yes
Feeding guild:			
Frugivorous	FRU	Binary	0. no, 1. Yes
Granivorous	GRN	Binary	0. no, 1. Yes
Herbivorous	HER	Binary	0. no, 1. Yes
Insectivorous	INS	Binary	0. no, 1. Yes
Omnivorous	OMN	Binary	0. no, 1. Yes
Vermivorous	VER	Binary	0. no, 1. Yes
Foraging substrate:			
Air	FAI	Binary	0. no, 1. Yes
Bark	FBA	Binary	0. no, 1. Yes
Ground	FGR	Binary	0. no, 1. Yes
Low canopy	FLC	Binary	0. no, 1. Yes
Upper canopy	FUC	Binary	0. no, 1. Yes
Feeding technique:			
Forager	FOR	Binary	0. no, 1. Yes
Gleaner	GLE	Binary	0. no, 1. Yes
Sallier	SAL	Binary	0. no, 1. Yes
Screener	SCR	Binary	0. no, 1. Yes
Territory size	TER	Quantitative	
Forest edge distance	FED	Ordinal	1. edge species, 2. edge and forest species, 3. forest species
Arrival from migration	AFM	Quantitative	Day (number of)

### Statistical Analysis

All statistical analyses were performed using version 2.15.2 of the R-language and environment for statistical computing with appropriate packages (R Core Team, Vienna, Austria). Deer densities (quantitative variable) and vegetation cover types (nominal variable) were used as explanatory variables in all analyses. Rare species were removed from the database. For plants, this corresponds to the species surveyed in less than 5% of the quadrats (n = 93). Rare ground beetles were those captured less than four times (n = 17) and rare songbird species (n = 7) were those surveyed in only one point-count.

**Figure 1:**
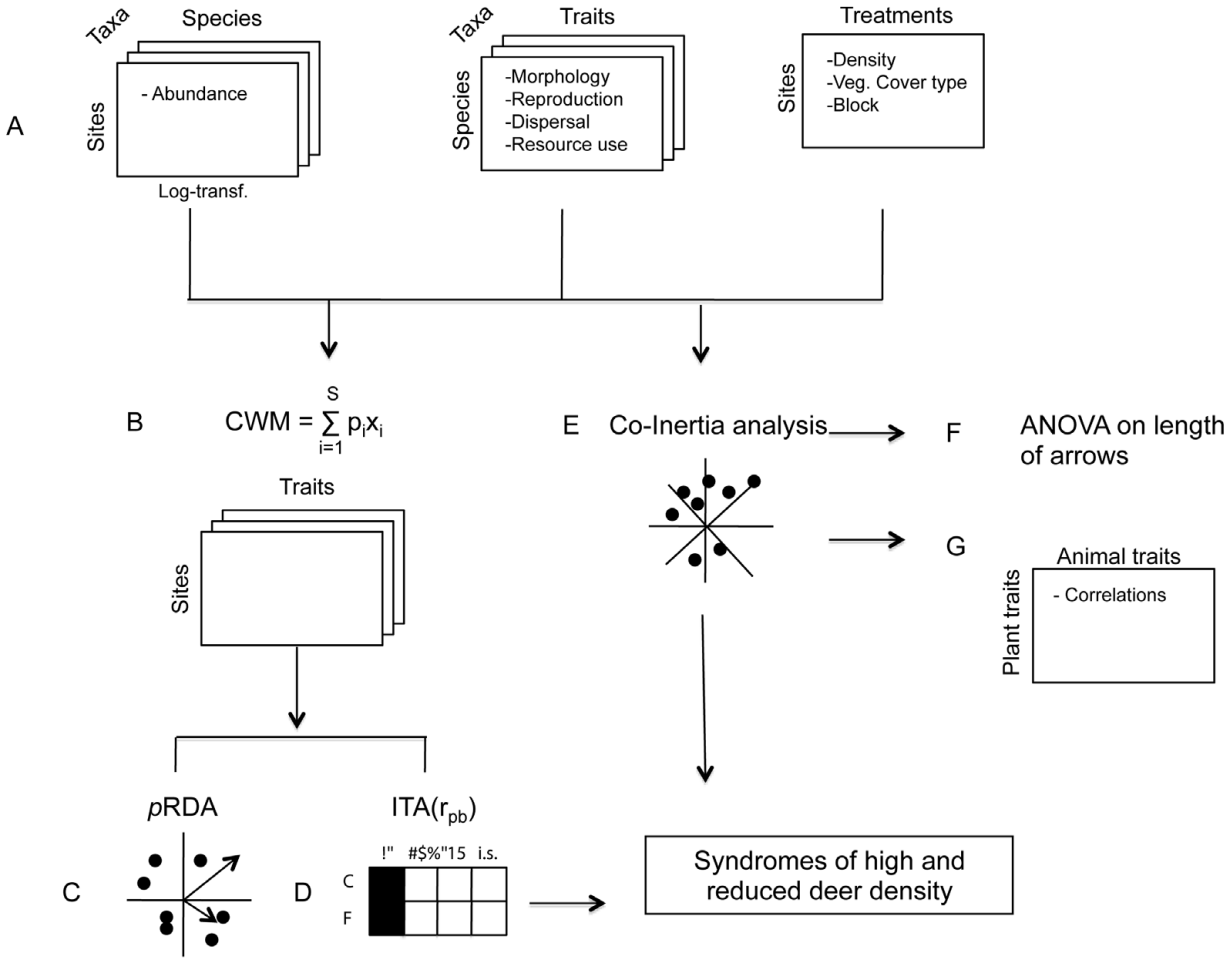
Diagram of the statistical approach. Sequence of the statistical analyses used to assess the functional syndromes of plant and animal traits associated with deer density. Distinct matrices were used for plants, ground beetles and songbirds (A). The matrix Treatment was the same for each group with the variables vegetation cover type (uncut forests and cut-over areas), deer density and block (A). The matrices CWM (community weighted mean) of traits by sites combine the Abundance (p_i_) and Trait (x_i_) matrices using weighted averaging (B). The partial redundancy analysis (*p*RDA; C), indicator traits analysis (ITA; D) and co-inertia analysis (E) were conducted with the CWM matrices. The impact of deer density on the degree of association of functional traits between two taxonomic groups (length of arrows in co-inertia graph) was tested with ANOVA (F). Finally, a correlation matrix between plant and animal traits was calculated (G)

First, we created both an abundance matrix and a trait matrix for each taxonomic group (Fig. 1A). We also built a treatment matrix with the combined treatment of deer density, vegetation cover type and blocks for each site. To quantify the trait values of the dominant species in a community, a Community Weighted Mean matrix (CWM; [34]) was calculated for each of the three groups by combining the species-by-sites matrix with the traits-by-species matrix (Fig. 1B). The weighted averaging procedure used the log (abundance +1) transformation to reduce the effects of extreme values [35], [36]. The CWM was then analyzed with the treatment matrix in a partial redundancy analysis (*p*RDA; [35], using block as a co-variable, to assess the effect of deer density under both types of vegetation cover (Fig. 1C).

We also carried out an indicator traits analysis (ITA; Fig. 1D) to identify traits associated with each possible combination of the eight experimental conditions, i.e., four classes of deer density, and two types of vegetation cover, for a total of 255 combinations (2^8^−1), including the set of eight conditions [37]. From the 255 combinations, we retained only the 54 combinations that could be interpreted ecologically (See Supporting Information Fig. S1). We used the point-biserial correlation coefficient (*r*_pb_) for this analysis because we were interested in the ecological preference associated to each trait [38]. A high *r*_pb_ value denotes a strong association between a trait and an experimental condition. A permutation test (*N* = 9999) was used to determine whether a trait was statistically associated with a combination of the experimental conditions under the null hypothesis of no relationship.

Co-inertia Analysis (CoIA; [39] was used to examine the association between plant and animal traits. We did one analysis using plants and ground beetle traits and another using plant and songbird traits (Fig. 1E). This analysis involved first reducing the dimensionality of the plant and animal CWM matrices using principal component analysis (PCA) and selecting the dominant components (axes). New axes are then generated by rotation in multidimensional space so as to maximize the covariance between the axes in the two datasets [39]. In the CoIA specific to plant and ground beetle traits, the selection of PCA axes included seven eigenvectors from the plant trait matrix and five eigenvectors from the ground beetle trait matrix, representing 88% and 86% of the variation, respectively. In the CoIA specific to plant and songbird traits, seven eigenvectors from the plant trait matrix and six eigenvectors were selected from PCA of the songbird traits matrix, representing 87% and 85% of the variation. For each CoIA, an RV coefficient (a multivariate generalization of the Pearson correlation coefficient) was then calculated as a measure of global correlation between the matrices. This coefficient ranges between 0 and 1; the closer it is to 1, the greater the global similarity between the two matrices. The significance of the association between the plant and animal traits was then tested with a Monte-Carlo permutation test using 9999 permutations. The results of CoIA are presented in a graph, with sites indicated as arrows, which in this case describe the degree of association of functional traits between two taxonomic groups (plants-ground beetles or plants-songbirds). Arrow length indicates the strength of the association between both matrices. Short arrows represent objects that are close in the space of the CoIA (strong relationships), whereas long arrows represent objects that are far away from each other's (weak relationships). To evaluate whether the degree of association between trait communities of two taxonomic groups was related to deer density and vegetation cover type, we used arrow length (calculated using the coordinates from the CoIA graph) as the dependent variable in an ANOVA, where deer density and vegetation cover type were the explanatory variables (Fig. 1F). Finally, we calculated the Pearson correlation coefficient between each plant and ground beetle trait and between each plant and songbird trait to determine which specific plant traits were linked with specific animal traits (Fig. 1G).

## Results

After discarding rare species, a total of 51 species of vascular plants, 13 species of ground beetles and 31 species of songbirds were used in the analysis.

**Figure 2:**
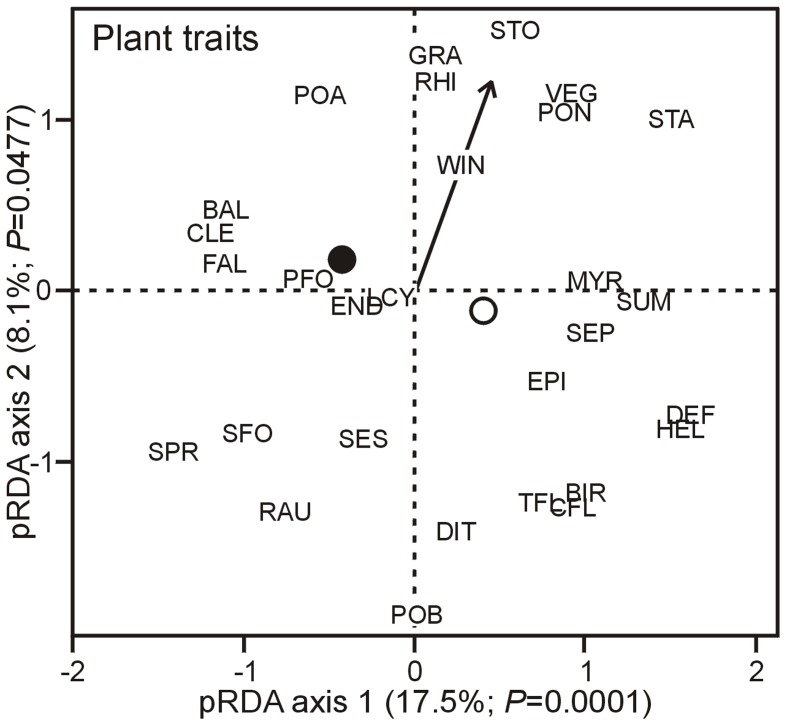
Plant trait response to deer density and vegetation cover type. Partial redundancy analysis showing the response of plant traits to deer density (arrow) and vegetation cover types (black circle  =  uncut forests; white circle  =  cut-over areas). Blocks were used as a co-variable. See Table 1 for trait names

The community of plant traits showed a clearer response than animal taxa to a reduction of deer density. More precisely, the impact of deer density was associated to the second axis of each of the three *p*RDA run with plant (Fig. 2), ground beetle and songbird traits (See Supporting Information Fig. S2), but it was significant only for plant traits (second axis significant with 8% variation explained mainly by deer density). For all three taxonomic groups, the vegetation cover type, whether uncut forest or cut-over area, was the main factor explaining trait distribution (first axis explaining 18% for plant traits, and 40% for both ground beetle and songbird traits).

### Plant Traits

**Figure 3:**
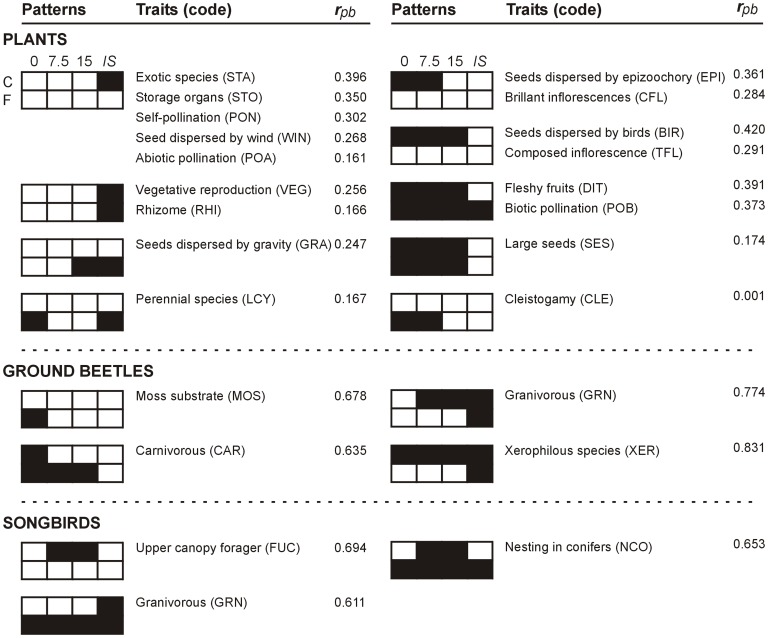
Traits indicators of deer density. Plant, ground beetle and songbird traits found to be indicators of at least one deer density experimental condition (0, 7.5 or 15 deer · km^−2^; *IS* =  *in situ* deer density, i.e., >27 deer · km^−2^). Black squares indicate significant positive correlations between the occurrence of a trait and a specific or several deer densities (*P*≤0.05). Point-biserial correlation coefficient (r_pb_) is indicated for each trait. Traits that only indicate a vegetation cover type (C =  cut-over areas; F =  uncut forests) are not presented

According to ITA and *p*RDA, seven plant traits were mainly associated with high deer density, especially *in situ* density, whether in cut-over areas or uncut forests or both (Figs 2 and 3). These traits corresponded to plant species that reproduce mainly vegetatively that have rhizomes or storage organs, that are self-pollinated, and which seeds and pollen grains dispersed abiotically. Exotic species were also associated to *in situ* deer density. On the other hand, eight plant traits were mostly associated with a reduction of deer density, mainly in cut-over areas (Fig. 3). These traits corresponded to plant species with brilliant and composed inflorescences, and dependent on biotic pollination. Species with fleshy fruits and large seeds dispersed by birds or by epizoochory (carried externally by animals) were also indicator of reduced deer density (RDD). According to ITA, cleistogamy (i.e., the presence of a closed and self-pollinated flower at the base of the plant) was associated with the two lowest deer densities (≤7.5 deer · km^−2^) in uncut forests (Fig. 3). Finally, according to *p*RDA, species with erected foliage or multi-stems (SFO), high Raunkiaer life forms (RAU), and flowering during spring (SPR) were also characteristics of reduced deer density (Fig. 2).

### Ground Beetle Traits

Ground beetle species using moss substrate were indicators of uncut forests from which deer had been completely eradicated (Fig. 3). Carnivorous species were associated to reduced deer densities (≤15 deer · km^−2^) in uncut forests and to the deer-eradicated cut-over areas. Granivorous species showed the exact opposite trend. Finally, xerophile species were indicators of *in situ* density in uncut forests, although these species were mostly associated with cut-over areas.

### Songbird Traits

The presence songbirds foraging in the upper canopy and nesting in conifers were indicator of intermediate deer density in cut-over areas (Fig. 3). Much as for ground beetles, granivorous bird species were associated with high deer densities, though only with *in situ* density of cut-over areas.

### Relationships between Plant and Animal Traits

**Figure 4:**
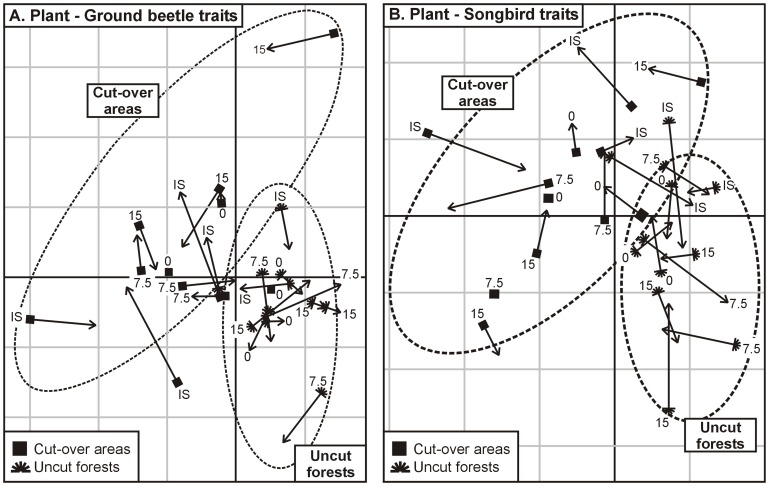
Relationship between plants and fauna traits after deer density reduction and logging. Co-inertia analyses comparing the distribution of plant and ground beetle (A) and plant and songbird (B) community trait compositions in the 24 experimental units (three blocks; four densities: 0, 7.5, 15 deer · km^−2^ and *in situ* (IS) density >27 deer · km^−2^; two vegetation cover types: uncut forests and cut-over areas). Arrows link plant and animal trait communities. The arrow tail represents the plant traits and the head the animal traits, while arrow length indicates the strength of the relationship between both matrices. Short arrows indicate a strong relationship, long arrows a weak relationship.

The association between plant and ground beetle traits was significant (RV = 0.41; *p* = 0.0004) for the first and second axes of the co-inertia graph (Fig. 4A). The first axis explained 66% of the variance and was mostly associated with the vegetation cover type, cut-over areas being mostly on the left side of the graph and uncut forests on the right (Fig. 4A). The second axis explained 13% of the variance and could not be clearly attributed to deer density or vegetation cover type, although most of the sites with low deer density (0 and 7.5 deer · km^−2^) occurred in the lower part of the graph. The strength of the association between plant and ground beetle community traits was significantly different between deer densities (Table 4), and increased as deer density decreased, as illustrated by the shorter arrows at low deer density than at high deer density (Fig. 4A). No effect of vegetation cover type was detected on the degree of association between traits communities of the plants and ground beetles (Table 4). Finally, plant traits associated with high deer density had few or no significant correlations with ground beetle traits, while most of the plant traits associated with reduced deer density were significantly correlated with ground beetle traits (See Supporting Information Table S1).

**Table 4:** Two-way analysis of variance (ANOVA) of the mean arrow length in the co-inertia analysis for the factors deer density (0, 7.5, 15 deer · km^−2^ and *in situ*: IS) and vegetation cover type (uncut forests and cut-over areas), and their interactions.

Plant – Ground beetle traits						
ANOVA				LSD test		
Variable	Df	*F*	*P*-value	Deer density	Mean row length	Groups
Deer density	1	4.670	0.043	0	0.6991	a
Vegetation cover type	1	0.067	0.798	7.5	0.9746	ab
Deer density*Cover type	1	1.812	0.193	15	0.9569	ab
Residuals	20			IS	1.232	b
				Least Significant Difference 0.479		
**Plant – songbird traits**						
Deer density	1	0.923	0.348	0	0.865	a
Vegetation cover type	1	1.402	0.250	7.5	1.074	a
Deer density*Cover type	1	0.619	0.440	15	1.027	a
Residuals	20			IS	0.865	a
				Least Significant Difference 0.444		

Deer densities with the same letter in the column groups have no significant difference in the arrow length.

The association between plant and songbird traits was also significant (RV = 0.43, p = 0.0004) for the first and second axes (Fig. 4B). Cut-over areas were separated from uncut forests along the first axis (67% of the variance explained). No obvious effect of deer density on the distribution of sites was recognizable in the co-inertia graph. The strength of the association between plant and songbird community traits was not significantly different between deer densities or vegetation cover types (Table 4).

## Discussion

Our study is the first to identify multi-taxa functional syndromes generated by deer density, and especially deer reduction, in forest ecosystems. As expected, we found evidence of a deer density syndrome in plants, and a weaker influence of deer reduction on ground beetles and songbirds. Similar low re-establishment of animal despite good re-establishment of plants, have been observed in other types of restored ecosystems, and have been mainly ascribed to the lack of heterogeneity in environmental conditions of restored sites despite vegetation recovery [40], [41]. We found a relatively low variance attributed to deer density (e.g., Fig.2). It may partially be explained by the filtering of the regional species pool by 75 years of deer overabundance, reducing the range of functional traits represented in the regional pool. As an example, the palatable woody characteristic plants of balsam fir stands such as *Corylus cornuta*, and *Taxus canadensis* have almost been extirpated [29], [42]. Some individuals remain but their reestablishment may take a more than the timeline of this study (6 years). It may also in part be explained by the use of categorical traits (Table 1–3) to describe communities, although categorical traits were coded as semi-quantitative traits when possible (e.g. Raunkier forms). Still, we observed in only six years of deer density control a shift in functional trait composition of the dominant plant species, suggesting that the resilience of the balsam fir stands on the Anticosti Island is not compromised.

The high deer density syndrome (HDD) found in plants includes species that reproduce vegetatively, as well as species with abiotic pollination and seed dispersal (small seeds dispersed by wind or gravity). These traits have previously been identified as an efficient strategy for ensuring persistence under foraging pressure by large herbivores (for example, [1], [24], [25]. As the boreal forest is known to be a semi-productive ecosystem [43], [44], this HDD syndrome can be associated, at least in part, to the stress-ruderal strategy within Grime's C-S-R theory [45]. Ruderal species, mostly grass and exotic plants, able to colonize disturbed ecosystems are indeed known to increase their fitness by using self-pollination and by producing a high number of small seeds [46], [47] that can easily disperse and colonize newly available sites. This is also consistent with observation that graminoid plants, known to be stress tolerant and rapid colonizers, and to have below ground reserves [48], thrive under the HDD conditions of our study site [27].

The reduced deer density (RDD) syndrome found in plants includes obligate seeders with brilliant flowers pollinated by animals and producing large seeds and fleshy fruits dispersed by birds or other animals. Plants under RDD therefore seem to have the ability to invest in reproductive structures instead of developing defenses against herbivores [49], [50]. The RDD syndrome found could thus be associated, at least in part, with the competitor strategy in Grime's C-S-R theory [45]. Competitive plants develop the ability to succeed in productive and undisturbed conditions where pollination by insects is an advantage [49]. Investment in attractive flowers that foster pollination by insects may indicate higher insect abundance, usually favored in stable environments [47], [51]. The RDD syndrome found also comprise species with erect foliage and high Raunkiaer life forms like phanerophyte, characteristics usually associated to competitive species [45].

Most of traits associated to RDD syndrome can be considered effect traits, since they likely contribute to a cascade effect that impacts other components of the ecosystem. This is supported in part by CoIA results, which showed that the association between plants and ground beetles was stronger as deer density decreased. At RDD, we found ground beetle species associated with moss substrates. Mosses may have been favored by reduced trampling [52] as well as by the more shaded and moist conditions [53] created by the abundance of phanerophytes on sites with RDD [15]. Moreover, plant RDD syndrome may have favored a more complex food chain, since we observed dominance of carnivorous ground beetles at RDD and granivorous ones at HDD, findings that concur with the fact that disturbances tend to affect carnivorous ground beetles more strongly than granivorous or omnivorous species [54], [55]. Although few traits of ground beetle communities responded to deer density reduction, they were traits clearly associated to vegetation changes, highlighting the importance of effect traits in trophic interactions.

Our results suggest that songbird trait communities were not globally influenced by the reduction in deer density. Only two functional traits were associated to vegetation at mid-deer density in cut-over areas. These traits were related to foraging and nesting behavior in the canopy and were thus associated to tall vegetation structure rather than to specific effect traits of plants in the understory. Deer browsing is known to strongly reduce the occurrence of bird species dependent on the understory in boreal forests [21], deciduous European woodlands [20], and in the temperate coastal forests of the Haida Gwaii archipelago in western Canada [56]. Although understory bird assemblages have been shown to recover successfully in deciduous forests [57], the boreal forest seems less resilient to long-term deer overpopulation. Even strategic logging to foster the regeneration of key tree species does not result in the return of a functional bird guild associated with the shrub layer [22]. As bird mobility is not constrained at the scale considered in this study (Venier and Aubin Unpublished Data), six years of regeneration seems insufficient to generate an understory dense and diverse enough to restore the bird traits community, despite the return of effect traits known to favor specific birds such as fleshy fruits.

## Conclusion

Six years of reduced deer density may not have been sufficient to allow the complete functional recovery of natural communities found in balsam fir stands after nearly 75 years of local over-browsing on Anticosti Island. However, our results, suggest that the boreal forest seemed able to recover some of the functional components fostering interactions between taxonomic groups within this short time period. Plant traits reappearing after deer density reduction were indeed correlated with ground beetle traits. The abundance of carnivorous ground beetles at low deer density is an additional indicator of the re-establishment of more complex trophic interactions. For songbirds, six years was likely too short to enable the recovery of a vegetation cover heterogeneous enough to attract naturally-associated understory birds. However, our approach can indicate changes in unstudied animal communities. Among others, pollinators are likely to have been favored at reduced deer densities as plants depending on biotic pollination for reproduction reappeared. Our study is one of the first multi-trophic investigations of forest resilience to high browsing intensity and was dedicated to a general understanding of the ecosystem capacity to recover from major disturbances. This approach could be complemented by a recent framework proposed by Lavorel et al. (2013) [2], which aims to identify and test key trait-based mechanisms delivering specific ecosystem services. The approach disentangles response traits from effect traits of two trophic levels in order to capture functional relationship driving trophic interactions. Combining such specific investigation of trophic interactions with comprehensive system analyses such as the one presented here should lead to a better understanding of ecosystem resilience after environmental changes.

## Supporting Information

Figure S1 **The 54 combinations used to identify indicator species of deer density (0, 7.5, 15 deer · km^−2^ and** ***in situ*** **(IS) density >27 deer · km^−2^) in two vegetation cover types (C =  cut-over areas; F  =  uncut forests).**
(DOC)

Figure S2 **Partial redundancy analysis showing the response of ground beetle and songbird traits to deer density (arrow) and vegetation cover types (black circle  =  uncut forests; white circle  =  cut-over areas).** Blocks were used as a co-variable. See Tables 2 and 3 for trait names.
(DOC)

Table S1 **Correlation matrix between plant and ground beetle traits.** Plant traits are presented in rows and ground beetle traits in columns. Rows in dark and blade gray are, respectively, plant traits associated to reduced and high deer density. See Tables 1 and 2 for code names.
(DOC)

## References

[1] Diaz S, Lavorel S, McIntyre S, Falczuk V, Casanoves F, et al (2007) Plant trait responses to grazing - a global synthesis. Glob Chang Biol 13: 313–341 10.1111/j.1365-2486.2006.01288.x

[2] Lavorel S, Storkey J, Bardgett RD, de Bello F, Berq M, et al (2013) A novel framework for linking functional diversity of plants with other trophic levels for the quantification of ecosystem services. J Veg Sci 24: 942–948 10.1111/jvs.12083

[3] Violle C, Navas ML, Vile D, Kazakou E, Fortunel C, et al (2007) Let the concept of trait be functional!. Oikos 116: 882–892 10.1111/j.2007.0030-1299.15559.x

[4] Demars B, Kemp J, Friberg N (2012) Linking biotopes to invertebrates in rivers: biological traits, taxonomic composition and diversity. Ecol Indic 23: 301–311 10.1016/j.ecolind.2012.04.011

[5] Pakeman RJ (2011) Multivariate identification of plant functional response and effect traits in an agricultural landscape. Ecology 92: 1353–1365 10.1890/10-1728.1 21797163

[6] de Bello F, Lepš J, Sebastià M (2005) Predictive value of plant traits to grazing along a climatic gradient in the Mediterranean. J Appl Ecol 42: 824–833 10.1111/j.1365-2664.2005.01079.x

[7] Moretti M, De Cáceres M, Pradella C, Obrist MK, Wermelinger B, et al (2010) Fire-induced taxonomic and functional changes in saproxylic beetle communities in fire sensitive regions. Ecography 33: 760–771 10.1111/j.1600-0587.2009.06172.x

[8] Kremen C (2005) Managing ecosystem services: what do we need to know about their ecology? Ecol Lett 8: 468–479 10.1111/j.1461-0248.2005.00751.x 21352450

[9] Moretti M, Legg C (2009) Combining plant and animal traits to assess community functional responses to disturbance. Ecography 32: 299–309 10.1111/j.1600-0587.2008.05524.x

[10] de Bello F, Lavorel S, Gerhold P, Reier Ü, Pärtel M (2010) A biodiversity monitoring framework for practical conservation of grasslands and shrublands. Biol Conserv 143: 9–17 10.1016/j.biocon.2009.04.022

[11] Luck G, Lavorel S, McIntyre S (2012) Improving the application of vertebrate trait-based frameworks to the study of ecosystem services. J Anim Ecol 81: 1065–1076 10.1111/j.1365-2656.2012.01974.x 22435774

[12] Suding KN, Lavorel S, Chapin III FS, Cornelissen JHC, Díaz S, et al (2008) Scaling environmental change through the community-level: a trait-based response-and-effect framework for plants. Glob Change Biol 14: 1125–1140 10.1111/j.1365-2486.2008.01557.x

[13] Côté SD, Rooney TP, Tremblay J-P, Dussault C, Waller DM (2004) Ecological impacts of deer overabundance. Annu Rev Ecol Evol Syst 35: 113–147 10.1146/annurev.ecolsys.35.021103.105725

[14] Husheer S, Coomes D, Robertson A (2003) Long-term influences of introduced deer on the composition and structure of New Zealand *Nothofagus* forests. For Ecol Manage 181: 99–117 10.1016/S0378-1127(03)00120-8

[15] Tremblay JP, Huot J, Potvin F (2007) Density-related effects of deer browsing on the regeneration dynamics of boreal forests. J Appl Ecol 44: 552–562 10.1111/j.1365-2664.2007.01290.x

[16] Augustine D, McNaughton S (1998) Ungulate effects on the functional species composition of plant communities: herbivore selectivity and plant tolerance. J Wildl Manage 62: 1165–1183 10.2307/3801981

[17] Hidding B, Tremblay JP, Côté SD (2012) Survival and growth of balsam fir seedlings and saplings under multiple controlled ungulate densities. For Ecol Manage 276: 96–103 10.1016/j.foreco.2012.03.023

[18] McInnes P, Naiman R, Pastor J, Cohen Y (1992) Effects of moose browsing on vegetation and litter of the boreal forest, Isle Royale, Michigan, USA. Ecology 73: 2059–2075 10.2307/1941455

[19] Dufresne M, Bradley R, Tremblay JP, Côté SD (2011) Evidence that soil depth and clay content control the post-disturbance regeneration of balsam fir and paper birch under heavy browsing from deer. Ecoscience 18: 363–368 10.2980/18-4-3366

[20] Perrins C, Overall R (2001) Effect of increasing numbers of deer on bird populations in Wytham Woods, central England. Forestry 74: 299–309 10.1093/forestry/74.3.299

[21] Cardinal É, Martin JL, Côté SD (2012) Large herbivore effects on songbirds in boreal forests: lessons from deer introduction on Anticosti Island. Ecoscience 19: 38–47 10.2980/19-1-3441

[22] Cardinal É, Martin JL, Tremblay JP, Côté SD (2012) An experimental study of how variation in deer density affects vegetation and songbird assemblages of recently harvested boreal forests. Can J Zool 90: 704–713 10.1139/Z2012-037

[23] Brousseau PM, Hébert C, Cloutier C, Côté SD (2013) Short-term effects of reduced white-tailed deer density on insect communities in a strongly overbrowsed boreal forest ecosystem. Biodivers Conserv 22: 77–92 10.1007/s10531-012-0400-5

[24] Wiegmann SM, Waller DM (2006) Fifty years of change in northern upland forest understories: Identity and traits of “winner” and “loser” plant species. Biol Conserv 129: 109–123 10.1016/j.biocon.2005.10.027

[25] Lavorel S, McIntyre S, Landsberg J, Forbes TDA (1997) Plant functional classifications: from general groups to specific groups based on response to disturbance. Trends Ecol Evol 12: 474–478 10.1016/S0169-5347(97)01219-6 21238163

[26] Lavorel S, McIntyre S, Grigulis K (1999) Plant response to disturbance in a Mediterranean grassland: How many functional groups? J Veg Sci 10: 661–672 10.2307/3237081

[27] Tremblay JP, Huot J, Potvin F (2006) Divergent nonlinear responses of the boreal forest field layer along an experimental gradient of deer densities. Oecologia 150: 78–88 10.1007/s00442-006-0504-2 16896768

[28] Beguin J, Prévost M, Pothier D, Côté SD (2009) Establishment of natural regeneration under severe browsing pressure from white-tailed deer after group seed-tree cutting with scarification on Anticosti Island. Can J For Res 39: 596–605 10.1139/X08-196

[29] Potvin F, Beaupré P, Laprise G (2003) The eradication of balsam fir stands by white-tailed deer on Anticosti Island, Québec: a 150-year process. Ecoscience 10: 487–495.

[30] Beaupré P, Bédard C, Dufour C, Gingras A, Potvin F, et al (2005) L'île d'Anticosti a son plan général d'aménagement intégré des ressources du milieu forestier. Nat Can 129: 110–117.

[31] Jobin LJ, Coulombe C (1992) The Luminoc® insect trap. Québec: Forestry Canada Information Leaflet LFC 26: 12 p..

[32] Hébert C, Jobin L, Fréchette M, Pelletier G (2000) An efficient pit-light trap to study beetle diversity. J Insect Conserv 4: 191–202 10.1023/A:1009611501133

[33] Bibby CJ, Burgess ND, Hill DA, Mustoe SH (2000) Bird census techniques. Second edition. London: Academic Press. 302p.

[34] Garnier E, Cortez J, Billes G, Navas M, Roumet C, et al (2004) Plant functional markers capture ecosystem properties during secondary succession. Ecology 85: 2630–2637 10.1890/03-0799

[35] Legendre P, Legendre L 1998. Numerical ecology. Second edition. Amsterdam: Elsevier. 853p.

[36] de Bello F, Lepš J, Lavorel S, Moretti M (2007) The importance of the species abundance on the measurement of trait composition: as example with pollinators' communities. Community Ecol 8: 163–170 10.1556/ComEc.8.2007.2.3

[37] De Cáceres M, Legendre P, Moretti M (2010) Improving indicator species analysis by combining groups of sites. Oikos 10: 1674–1684 10.1111/j.1600-0706.2010.18334.x

[38] De Cáceres M, Legendre P (2009) Associations between species and groups of sites: indices and statistical inference. Ecology 90: 3566–3574 10.1890/08-1823.1 20120823

[39] Doledec S, Chessel D (1994) Co-inertia analysis: an alternative method for studying species-environment relationships. Freshwater biol 31: 277–294 10.1111/j.1365-2427.1994.tb01741.x

[40] Desrochers A, Rochefort L, Savard JPL (1998) Avian recolonization of Eastern Canadian bogs after peat mining. Can J Zool 76: 989–997 10.1139/cjz-76-6-989

[41] Jansen A (2005) Avian use of restoration plantings along a creek linking rainforest patches on the Atherton Tablelands, North Queensland. Restor Ecol 13: 275–283 10.1111/j.1526-100X.2005.00035.x

[42] Pimlott DH (1963) Influence of deer and moose on boreal forest vegetation in two areas of Eastern Canada. Int. Union Game Biol. Congr. 6: 106–116.

[43] Whittaker RH (1975) Communities and ecosystems. Second edition. New York: Macmillan Publishing. 383p.

[44] Malhi Y, Baldocchi DD, Jarvis PG (1999) The carbon balance of tropical, temperate and boreal forests. Plant Cell Environ 22: 715–740 10.1046/j.1365-3040.1999.00453.x

[45] Grime JP (2001) Plant strategies, vegetation processes and ecosystem properties. Chichester: John Wiley & Sons Ltd. 456p.

[46] Baker HG (1972) Seed weight in relation to environmental conditions in California. Ecology 53: 997–1010 10.2307/1935413

[47] Šerá B, Šerý M (2004) Number and weight of seeds and reproductive strategies of herbaceous plants. Folia Geobotanica 39: 27–40 10.1007/BF02803262

[48] Mulder CPH (1999) Vertebrate herbivores and plants in the Arctic and subarctic: effects on individuals, populations, communities and ecosystems. Perspect Plant Ecol Evol Syst 2: 29–55 10.1078/1433-8319-00064

[49] Grime JP (1977) Evidence for the existence of three primary strategies in plants and its relevance to ecological and evolutionary theory. Am Nat 111: 1169–1194.

[50] Herms DA, Mattson WJ (1992) The dilemma of plants: to grow or defend. Quart Rev Biol 67: 283–335.

[51] Kevan PG, Baker HG (1983) Insects as flower visitors and pollinators. Annu Rev Entomol 28: 407–453 10.1146/annurev.en.28.010183.002203

[52] Van Der Wal R, Brooker R (2004) Mosses mediate grazer impacts on grass abundance in arctic ecosystems. Funct Ecol 18: 77–86 10.1111/j.1365-2435.2004.00820.x

[53] Gardner S, Hartley S, Davies A, Palmer S (1997) Carabid communities on heather moorlands in northeast Scotland: the consequences of grazing pressure for community diversity. Biol Conserv 81: 275–286 10.1016/S0006-3207(96)00148-6

[54] Barbaro L, van Halder I (2009) Linking bird, carabid beetle and butterfly life-history traits to habitat fragmentation in mosaic landscapes. Ecography 32: 321–333 10.1111/j.1600-0587.2008.05546.x

[55] Barton PS, Manning AD, Gibb H (2011) Experimental reduction of native vertebrate grazing and addition of logs benefit beetle diversity at multiple scales. J Appl Ecol 48: 943–951 10.1111/j.1365-2664.2011.01994.x

[56] Allombert S, Gaston A, Martin JL (2005) A natural experiment on the impact of overabundant deer on songbird populations. Biol Conserv 126: 1–13 10.1016/j.biocon.2005.04.001

[57] McShea W, Rappole J (2000) Managing the abundance and diversity of breeding bird populations through manipulation of deer populations. Conserv Biol 14: 1161–1170 10.1046/j.1523-1739.2000.99210.x

