# Supplementary material for: Functional Responses and Resilience of Boreal Forest Ecosystem after Reduction of Deer Density
Source: PLoS One. 2014 Feb 28;9(2):e90437. doi: 10.1371/journal.pone.0090437 (PMC3938752; doi:10.1371/journal.pone.0090437)
Supplement: Figure S1 — The 54 combinations used to identify indicator species of deer density (0, 7.5, 15 deer · km−2 and in situ (IS) density >27 deer · km−2) in two vegetation cover types (C = cut-over areas; F = uncut forests). (DOC) [file pone.0090437.s001.doc]

| 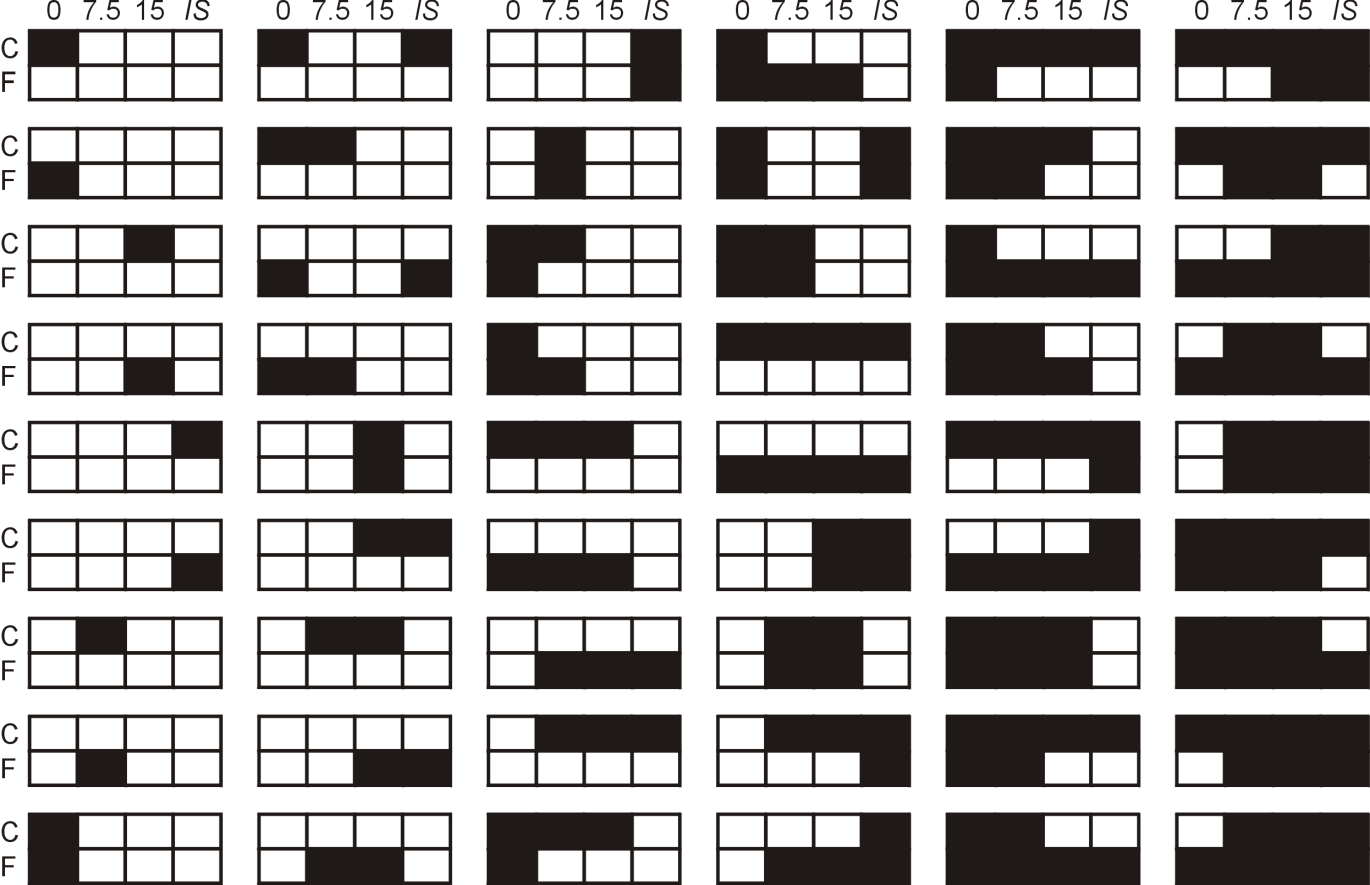 |
| --- |

**Figure S1** The 54 combinations used to identify indicator species of deer density (0, 7.5, 15 deer · km^-2^ and *in situ* (IS) density >27 deer · km^-2^) in two vegetation cover types (C = cut‑over areas; F = uncut forests)
