# Supplementary material for: Functional Responses and Resilience of Boreal Forest Ecosystem after Reduction of Deer Density
Source: PLoS One. 2014 Feb 28;9(2):e90437. doi: 10.1371/journal.pone.0090437 (PMC3938752; doi:10.1371/journal.pone.0090437)
Supplement: Figure S2 — Partial redundancy analysis showing the response of ground beetle and songbird traits to deer density (arrow) and vegetation cover types (black circle = uncut forests; white circle = cut-over areas). Blocks were used as a co-variable. See Tables 2 and 3 for trait names. (DOC) [file pone.0090437.s002.doc]

| 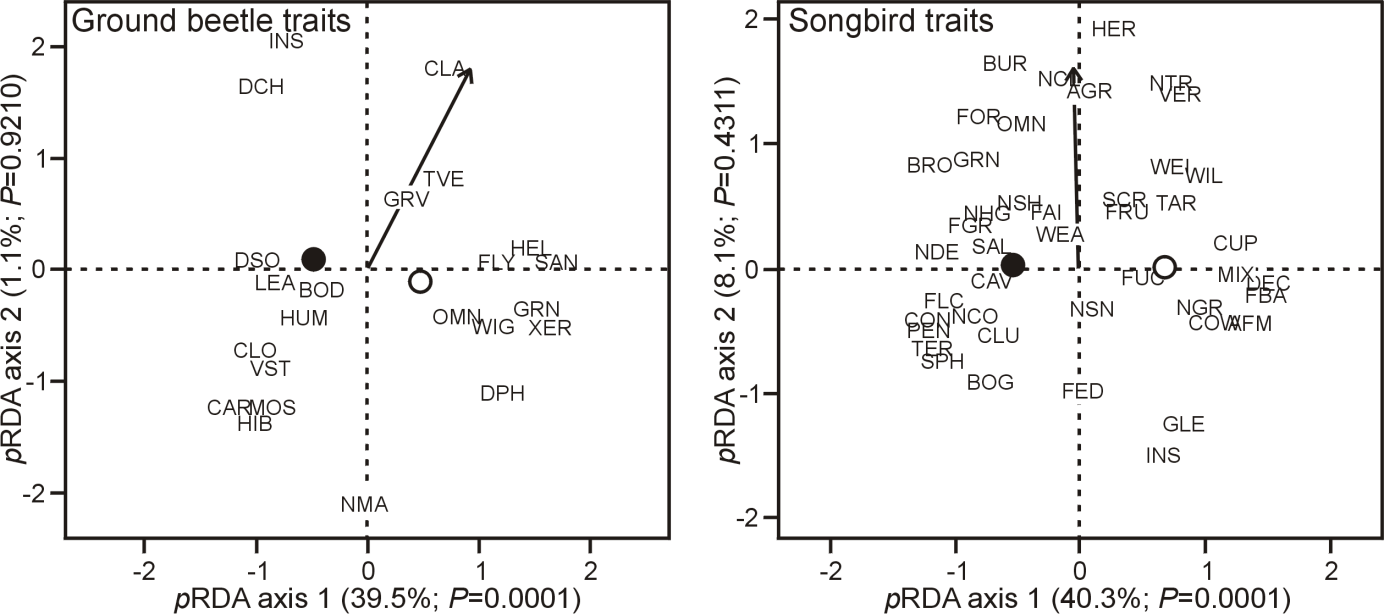 |
| --- |

**Figure S2** Partial redundancy analysis showing the response of ground beetle and songbird traits to deer density (arrow) and vegetation cover types (black circle = uncut forests; white circle = cut-over areas). Blocks were used as a co-variable. See Tables 2 and 3 for trait names
