# Supplementary material for: Functional Responses and Resilience of Boreal Forest Ecosystem after Reduction of Deer Density
Source: PLoS One. 2014 Feb 28;9(2):e90437. doi: 10.1371/journal.pone.0090437 (PMC3938752; doi:10.1371/journal.pone.0090437)
Supplement: Table S1 — Correlation matrix between plant and ground beetle traits. Plant traits are presented in rows and ground beetle traits in columns. Rows in dark and blade gray are, respectively, plant traits associated to reduced and high deer density. See Tables 1 and 2 for code names. (DOC) [file pone.0090437.s003.doc]

**Table S1** Correlation matrix between plant and ground beetle traits. Plant traits are presented in rows and ground beetle traits in columns. Rows in dark and blade gray are, respectively, plant traits associated to reduced and high deer density. See Tables 1 and 2 for code names

|  | **BOD** | **NMA** | **OMN** | **CAR** | **INS** | **GRN** | **VST** | **WIG** | **FLY** | **HIB** | **DPH** | **DCH** | **DSO** | **HEL** | **XER** | **CLO** | **TVE** | **SAN** | **GRV** | **CLA** | **HUM** | **LEA** | **MOS** |
| --- | --- | --- | --- | --- | --- | --- | --- | --- | --- | --- | --- | --- | --- | --- | --- | --- | --- | --- | --- | --- | --- | --- | --- |
| **PFO** | -0.06 | -0.05 | 0.19 | 0.01 | 0.01 | -0.05 | 0.19 | **-0.41** | -0.17 | 0.01 | -0.08 | 0.08 | 0.06 | **-0.45** | 0.01 | 0.39 | 0.27 | -0.32 | **-0.61** | -0.28 | **0.60** | **0.42** | -0.22 |
| **SFO** | -0.04 | 0.25 | 0.16 | 0.23 | 0.05 | -0.29 | 0.39 | -0.35 | -0.19 | 0.25 | -0.08 | 0.07 | 0.11 | **-0.57** | -0.19 | 0.38 | -0.04 | **-0.45** | -0.32 | **-0.44** | **0.47** | **0.43** | -0.05 |
| **LCY** | 0.13 | 0.06 | -0.04 | 0.04 | -0.26 | 0.16 | 0.00 | -0.15 | -0.01 | 0.12 | 0.04 | -0.05 | 0.03 | 0.05 | 0.08 | -0.06 | 0.27 | 0.05 | -0.10 | 0.17 | -0.16 | 0.09 | -0.11 |
| **DEF** | -0.13 | 0.03 | 0.13 | -0.40 | -0.28 | **0.56** | -0.34 | **0.49** | **0.41** | -0.23 | 0.35 | -0.33 | -0.34 | **0.54** | **0.49** | **-0.45** | 0.13 | **0.65** | 0.36 | 0.27 | **-0.41** | **-0.55** | -0.22 |
| **RAU** | 0.04 | -0.03 | 0.15 | 0.23 | -0.01 | -0.24 | 0.18 | -0.38 | -0.24 | 0.20 | -0.17 | 0.14 | 0.25 | **-0.50** | -0.20 | **0.41** | 0.13 | -0.39 | **-0.46** | -0.32 | 0.51 | 0.39 | -0.00 |
| **RHI** | -0.25 | -0.26 | 0.03 | -0.21 | 0.27 | -0.01 | **-0.41** | 0.15 | 0.19 | -0.36 | -0.05 | 0.09 | -0.19 | 0.01 | 0.07 | 0.14 | -0.01 | 0.00 | 0.05 | 0.18 | -0.04 | -0.08 | 0.12 |
| **STO** | -0.08 | -0.27 | -0.01 | -0.19 | 0.21 | 0.02 | -0.37 | 0.30 | 0.16 | -0.36 | -0.00 | 0.04 | -0.20 | 0.29 | 0.06 | -0.17 | -0.11 | 0.24 | 0.24 | 0.31 | -0.23 | -0.30 | 0.05 |
| **VEG** | -0.20 | 0.06 | 0.33 | -0.33 | -0.31 | **0.47** | -0.18 | 0.08 | 0.25 | -0.30 | 0.31 | -0.30 | -0.28 | 0.13 | **0.53** | -0.03 | 0.37 | 0.32 | -0.24 | 0.22 | 0.05 | -0.13 | -0.35 |
| **SPR** | 0.00 | 0.13 | 0.11 | **0.45** | 0.21 | **-0.60** | 0.31 | **-0.48** | **-0.42** | **0.42** | **-0.40** | 0.37 | **0.45** | **-0.66** | **-0.49** | **0.60** | -0.12 | **-0.63** | -0.11 | **-0.64** | 0.15 | **0.57** | 0.31 |
| **SUM** | 0.17 | 0.09 | -0.24 | -0.22 | -0.33 | **0.50** | -0.15 | **0.46** | 0.25 | -0.11 | 0.37 | -0.35 | -0.36 | **0.72** | 0.36 | **-0.67** | -0.01 | **0.60** | 0.27 | **0.47** | -0.37 | **-0.52** | -0.18 |
| **FAL** | **0.44** | 0.02 | **-0.53** | **0.50** | 0.12 | **-0.44** | 0.25 | -0.36 | -0.51 | **0.42** | **-0.45** | **0.45** | 0.27 | -0.05 | **-0.50** | 0.05 | -0.16 | -0.39 | -0.01 | -0.14 | -0.24 | **0.43** | 0.29 |
| **TFL** | 0.15 | 0.07 | 0.02 | 0.10 | **-0.45** | 0.24 | -0.36 | 0.11 | 0.04 | 0.12 | 0.03 | -0.04 | 0.03 | 0.28 | 0.16 | -0.09 | 0.24 | 0.29 | 0.16 | 0.15 | **-0.59** | -0.13 | 0.15 |
| **CFL** | 0.17 | 0.04 | -0.08 | 0.03 | **-0.53** | 0.38 | -0.22 | 0.01 | -0.03 | 0.03 | 0.19 | -0.19 | -0.11 | 0.34 | 0.33 | -0.11 | 0.35 | 0.25 | -0.26 | 0.28 | -0.17 | -0.04 | -0.07 |
| **CLE** | -0.08 | -0.22 | -0.29 | 0.04 | **0.67** | **-0.47** | **0.49** | -0.14 | -0.21 | 0.01 | -0.21 | 0.18 | 0.31 | -0.27 | **-0.48** | 0.12 | **-0.47** | **-0.48** | -0.04 | -0.29 | **0.62** | 0.14 | 0.17 |
| **POA** | 0.12 | -0.17 | -0.05 | 0.25 | 0.00 | -0.22 | -0.14 | -0.07 | -0.04 | 0.23 | -0.28 | 0.25 | 0.37 | -0.10 | -0.32 | 0.09 | 0.02 | -0.11 | 0.23 | 0.05 | -0.26 | 0.01 | 0.30 |
| **POB** | 0.06 | 0.22 | 0.03 | 0.13 | -0.28 | 0.09 | 0.15 | -0.24 | -0.19 | 0.18 | 0.06 | -0.07 | 0.02 | -0.13 | 0.12 | 0.20 | 0.20 | -0.13 | -0.38 | -0.22 | 0.16 | 0.29 | -0.06 |
| **PON** | -0.08 | -0.21 | 0.06 | -0.34 | 0.21 | 0.15 | -0.31 | 0.37 | 0.20 | -0.50 | 0.12 | -0.07 | -0.36 | **0.41** | 0.21 | -0.33 | -0.12 | 0.40 | 0.24 | 0.33 | -0.25 | -0.37 | -0.13 |
| **SEP** | 0.19 | 0.02 | -0.11 | -0.03 | -0.20 | 0.20 | -0.27 | 0.40 | 0.23 | -0.03 | 0.14 | -0.12 | -0.19 | **0.52** | 0.09 | **-0.5** | -0.08 | **0.49** | **0.52** | **0.50** | **-0.62** | **-0.48** | -0.00 |
| **WIN** | 0.10 | -0.32 | -0.18 | -0.11 | 0.24 | -0.03 | -0.16 | 0.21 | 0.08 | -0.24 | -0.01 | 0.03 | -0.13 | 0.25 | -0.09 | -0.30 | -0.17 | 0.14 | 0.11 | 0.36 | 0.04 | -0.26 | -0.03 |
| **END** | 0.08 | 0.21 | -0.21 | 0.08 | 0.21 | -0.19 | **0.47** | 0.12 | -0.02 | 0.29 | 0.02 | -0.03 | 0.04 | 0.03 | -0.27 | -0.31 | **-0.45** | -0.04 | **0.44** | -0.22 | 0.02 | -0.13 | -0.04 |
| **EPI** | 0.09 | 0.07 | -0.17 | 0.02 | -0.23 | 0.19 | -0.30 | 0.25 | 0.23 | 0.02 | 0.13 | -0.10 | -0.25 | 0.33 | 0.12 | -0.27 | 0.01 | 0.25 | 0.29 | **0.49** | **-0.43** | -0.25 | 0.04 |
| **MYR** | -0.12 | 0.24 | 0.14 | -0.11 | -0.35 | 0.33 | -0.38 | 0.41 | 0.51 | -0.04 | 0.28 | -0.25 | -0.33 | 0.24 | 0.29 | -0.25 | 0.05 | **0.41** | **0.43** | **0.47** | **-0.44** | **-0.44** | -0.04 |
| **BIR** | 0.12 | **0.44** | 0.14 | 0.00 | **-0.56** | 0.38 | 0.03 | 0.20 | 0.15 | 0.15 | 0.35 | -0.34 | -0.24 | 0.24 | 0.33 | -0.31 | 0.11 | 0.39 | 0.11 | 0.06 | -0.28 | -0.21 | -0.23 |
| **ANT** | **0.41** | -0.35 | 0.01 | **0.44** | -0.06 | -0.37 | -0.08 | -0.34 | **-0.42** | 0.32 | **-0.58** | **0.55** | **0.51** | -0.15 | **-0.45** | 0.14 | 0.19 | -0.16 | -0.03 | -0.09 | -0.22 | 0.25 | 0.16 |
| **BAL** | -0.26 | -0.12 | -0.09 | 0.00 | **0.68** | **-0.49** | 0.20 | -0.15 | -0.15 | -0.15 | -0.25 | 0.25 | 0.15 | -0.35 | -0.33 | 0.38 | -0.37 | **-0.48** | 0.00 | -0.31 | 0.26 | 0.26 | 0.28 |
| **GRA** | -0.31 | -0.15 | 0.36 | -0.18 | -0.05 | 0.12 | -0.34 | -0.09 | 0.09 | -0.23 | -0.03 | 0.03 | 0.00 | -0.20 | 0.24 | **0.42** | 0.34 | -0.02 | -0.25 | -0.08 | 0.06 | 0.12 | 0.01 |
| **SES** | 0.10 | 0.33 | -0.04 | 0.28 | -0.17 | -0.13 | 0.28 | -0.10 | -0.15 | **0.44** | -0.03 | 0.00 | 0.21 | -0.18 | -0.17 | 0.10 | -0.11 | -0.17 | 0.07 | -0.39 | -0.01 | 0.14 | 0.16 |
| **DIT** | 0.04 | 0.39 | 0.21 | 0.12 | **-0.50** | 0.22 | 0.12 | -0.16 | -0.02 | 0.29 | 0.16 | -0.17 | -0.02 | -0.13 | 0.22 | 0.11 | 0.29 | 0.03 | -0.19 | -0.19 | -0.02 | 0.17 | -0.17 |
| **HEL** | -0.07 | 0.06 | 0.21 | -0.21 | **-0.48** | **0.50** | -0.62 | **0.45** | 0.38 | -0.23 | 0.30 | -0.29 | -0.26 | **0.48** | **0.47** | -0.24 | 0.24 | **0.61** | 0.26 | **0.41** | **-0.62** | **-0.46** | 0.04 |
| **STA** | 0.06 | -0.32 | 0.05 | -0.32 | -0.17 | **0.41** | **-0.45** | 0.31 | 0.18 | **-0.42** | 0.17 | -0.14 | -0.27 | **0.61** | 0.38 | **-0.43** | 0.24 | **0.59** | 0.08 | **0.58** | -0.35 | **-0.43** | -0.23 |
